# Identification of circulating CD31^+^CD45^+^ cell populations with the potential to differentiate into erythroid cells

**DOI:** 10.1186/s13287-021-02311-y

**Published:** 2021-04-13

**Authors:** Maria Chiara G. Monaco, Dragan Maric, Ombretta Salvucci, Cristina Antonetti Lamorgese Passeri, Patrizia Accorsi, Eugene O. Major, Anna Concetta Berardi

**Affiliations:** 1 Viral Immunology Section, National Institute of Neurological, Disorders and Stroke, National Institutes of Health, Bethesda, MD USA; 2 NINDS Flow Cytometry Core Facility, National Institutes of Health, Bethesda, MD USA; 3 Laboratory of Cellular Oncology, Center for Cancer Research, National Cancer Institute, National Institutes of Health, Bethesda, MD USA; 4 Department of Haematology, Laboratory of Stem Cells, Transfusion Medicine and Biotechnologies, Santo Spirito Hospital, Pescara, Italy; 5 Department of Haematology, Transfusion Medicine and Biotechnologies, Santo Spirito Hospital, 65125 Pescara, Italy; 6 Laboratory of Molecular Medicine and Neuroscience, National Institute of Neurological Disorders and Stroke, National Institutes of Health, Bethesda, MD USA

**Keywords:** Erythro-myeloid progenitors, CD31^+^CD45^+^ circulating cells, Human erythropoiesis

## Abstract

Erythro-myeloid progenitors (EMP) are found in a population of cells expressing CD31 and CD45 markers (CD31^+^CD45^+^). A recent study indicated that EMPs persist until adulthood and can be a source of endothelial cells. We identified two sub-populations of EMP cells, CD31^low^CD45^low^ and CD31^high^CD45^+^, from peripheral blood that can differentiate into cells of erythroid lineage. Our novel findings add to the current knowledge of hematopoietic lineage commitment, and our sequential, dual-step, in vitro culture model provides a platform for the study of the molecular and cellular mechanisms underlying human hematopoiesis and erythroid differentiation.

**Supplementary Information:**

The online version contains supplementary material available at 10.1186/s13287-021-02311-y.

To the editor

Evolving views of hematopoietic stem and progenitor cell (HSPC) differentiation highlight complex regulation of lineage progression from highly heterogeneous HSPC populations [1]. Understanding the dynamic evolution of HSPCs will shed further light on erythropoiesis. HSPCs are generated from a specialized population of endothelial cells, called hemogenic cells, in the aorta-gonad-mesonephros (AGM) region, through an endothelial-to-hematopoietic transition (EHT) [2]. During embryogenesis, transient primitive hematopoiesis occurs in the yolk-sac, followed by another transient hematopoietic process, which starts from the erythro-myeloid progenitors (EMPs), the earliest precursors of erythrocytes, megakaryocytes, and macrophages, which originate in the yolk sac and migrate to the fetal liver [3]. After birth, human erythropoiesis occurs in the bone marrow in specialized hematopoietic stem cell niches enriched in mesenchymal cells and other stromal cells. EMPs are found in a population of cells expressing CD31 and CD45 markers (CD31^+^CD45^+^) [3, 4]. A recent study indicated that EMPs persist until adulthood, and were found to be a source of endothelial cells [5]. Baumann et al. demonstrated that CD31(CD31/PECAM-1) is expressed on mouse HSCs throughout ontogeny and an erythroid progenitor cell population subset persists in adult bone marrow [6].

To evaluate whether some human erythrocytes in the adult could arise from existing CD31^+^CD45^+^ circulating cells, we used a Lin^−^-enriched population derived from human peripheral blood (Fig. 1a; supplemental material) to identify other cell surface markers that correlate with CD31^+^CD45^+^ expression in adult peripheral blood. Our aim was to achieve a prospective characterization of CD31^+^CD45^+^ cell populations derived from Lin^−^ cells. A flow cytometry analysis of the CD31^+^CD45^+^ Lin^−^ cell population identified two discernible cell populations: CD31^low^ and CD31^high^ co-expressing CD45^low^ and CD45^+^, respectively (Fig. 1b). CD31^+^CD45^+^ cell populations are thought to be EMP progeny [3–5]. Furthermore, the analyses revealed a CD31^−^CD45^+^ cell population that we decided to use as a control (CTRL). We further characterized CD31^low^CD45^low^ (29%) and CD31^high^CD45^+^ (12%) cell populations, and the control cell population (37.3%) (Fig. 1c; Additional file 1) by screening antibodies against these markers and other markers relevant to HSPCs and erythropoietic differentiation. These include CD34 marker for the enrichment of HSPCs, CD117 (c-Kit) expressed on hematopoietic stem/progenitor cells and at the earliest pro-erythroblast stage; CD150 (SLAM; signaling lymphocyte activation molecule) known to mark megakaryocytic/erythroid progenitors during hematopoietic homeostasis, CD133 markers for the enrichment of HSPCs, and CD38, for a recently reported megakaryocyte/erythroid progenitor cell subpopulation residing in the CD38^mid^ cell population. Flow cytometry analysis confirmed that hematopoietic cells in the CD31^low^CD45^low^ population expressed CD117 (4.16%), CD34 (17.49%), CD150 (2.17%), but that CD133 expression was low (0.12%), while the majority of cells were CD38^mid^ (40.29%) (Fig. 1d and b panels CD31^low^CD45^low^;). In comparison to the CD31^low^CD45^low^ cell population, the hematopoietic cells in the CD31^high^CD45^+^ populations expressed very low CD117 (0.86%), CD34 (0.50%), CD150 (0.49%) and CD133 (0.18%), and 7.79% expressed CD38^mid^(Fig. 1d and b panels CD31^high^CD45^+^). The CTRL cell population expressed CD117 (4.7%), CD34 (0.06%), CD150 (0.03%), CD133(0.08%) and CD38^mid^ (20.57%) (Fig. 1d; Additional file 1). These data confirm the previous findings of a high heterogeneity within hematopoietic populations [7]. Previous in vitro studies support the finding that the majority of cells capable of differentiating into erythroblasts reside in the CD34^−^ cell population present in PBMCs and that these CD34^−^ cells first acquire expression of the CD34 marker before differentiating into erythroid lineage cells [8]. In addition, Sass et al. demonstrated that human CD31^+^ cells from peripheral blood are capable of differentiating into distinct cell lineages depending on the environmental cell population, suggesting cell-to-cell stimulation within these CD31^+^ cell subpopulations [9].

**Fig. 1:**
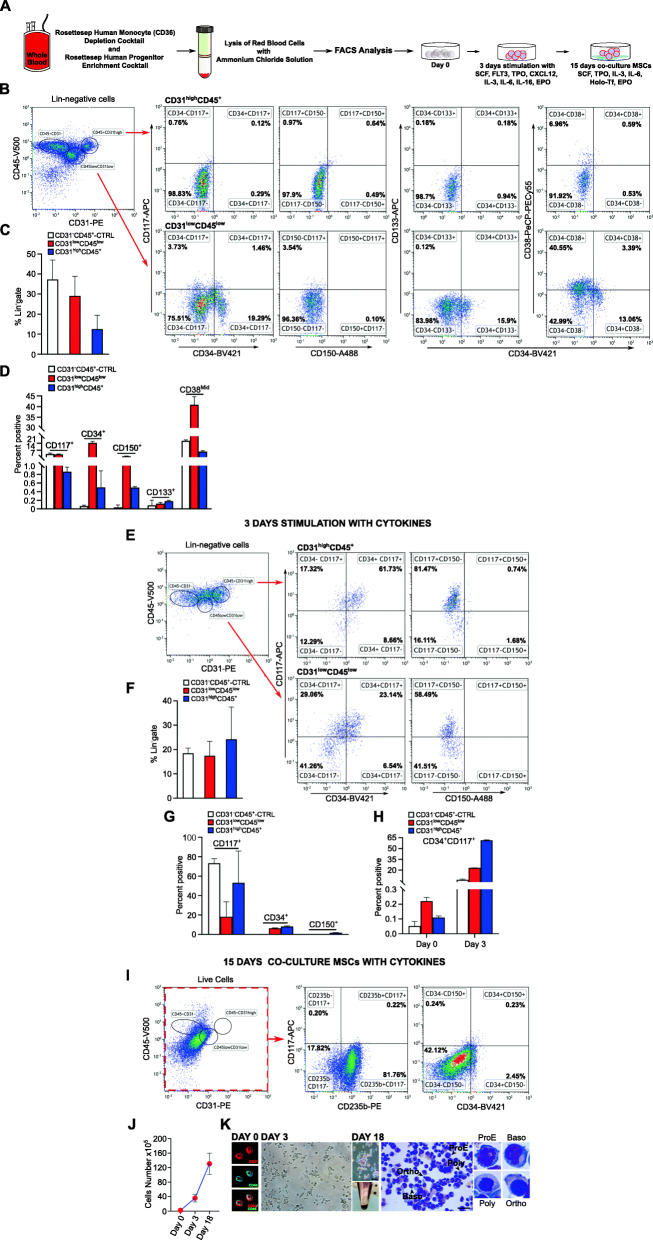
Flow cytometric characterization of circulating CD31^high^CD45^+^ and CD31^low^CD45^+^ cells in human peripheral blood. **a** Strategy for the isolation of Lin^−^ cells from “whole blood” for sequential cytokine stimulation and bone marrow mesenchymal stromal cell co-culture to promote erythroblast differentiation. **b** Represenative flow cytometric analysis of fresh adult human Lin^−^ CD31^high^CD45^+^ and CD31^low^CD45^low^ cell populations from enriched hematopoietic Lin^−^ cells from peripheral blood. **c** Frequency of CD31^+^ and CD45^+^ cells (bar graph). The total percentage of CD31^high^CD45^+^ and CD31^low^CD45^low^ cells and of the control CD31^−^CD45^+^ population (from the entire Lineage-negative cell population) are displayed as mean of *n* = 3 independent experiments ± SD. **d** Mean percentage of CD117^+^, CD34^+^, CD150^+^, and CD133^+^ cells ± SD are shown; *n* = 3 independent experiments. **e** Representative plots showing the distribution of CD31^high^CD45^+^ and CD31^low^CD45^low^ cell populations and immunophenotypic changes after 3 days of cytokine stimulation. **f** Cumulative data are shown as means ± SD of 3 independent experiments in the bar graph. Suspended and semi-adherent hematopoietic cells were collected for flow cytometry analysis. **g** Mean percentage of CD117^+^, CD34^+^, and CD150^+^ cells ± SD are shown; three independent experiments. **h** Mean percentage of ±SD CD34^+^CD117^+^ cells detected on day 0 and after 3 days of cytokine stimulation ± SD are shown; three independent experiments. **i** Representative flow cytometric analysis from the entire Lineage-negative population after 3 days of cytokine stimulation and 15 days of co-cultivation with MSCs. The cells lost CD31^high^CD45^+^ and CD31^low^CD45^low^ expression and acquired CD235b^+^ expression erythroid cells; *n* = 3 independent experiments. **j** Cell yields at the indicated time points (left panel). **k** The left panel shows representative fluorescence imaging (CD31 and CD45 markers) from the entire Lineage-negative cell population at day 0 of culture and bright-field microscopy images (original magnification 40×) after cytokine stimulation for 3 days; cells were stained with Wright-Giemsa at day 18 (after the 3 days of cytokine stimulation and the 15 days of co-culture on MSCs). Scale bars, 20 μm. The right panel shows representative images of basophilic erythroblasts (Baso), polychromatic erythroblasts (Poly), orthochromatic normoblasts (Ortho), on day 18 (3 + 15 days) after erythroid differentiation with cytokines and bone marrow mesenchymal stem cell co-culture; *n* = 3 independent experiments. Data are shown as means ± SD

To test this possibility, we proceeded to use the entire lineage-negative cell population to study the differentiation/maturation of CD31^low^CD45^low^ and CD31^high^CD45^+^ cells into erythroid lineage cells [9, 10]. Based on previous studies [9, 10], we assessed the first step of differentiation by culturing cells for 3 days in the presence of a cocktail of cytokines, including SCF, FLT3, TPO, CXCL12 (previously known as stromal cell-derived factor 1/SDF-1), IL-3, IL-6, and EPO. Flow cytometry analysis showed that the CD31^low^CD45^low^ cell population decreased during culture from 29 to 17% and the CD31^high^CD45^+^ cell population increased during culture from 12 to 24% (Fig. 1c, e, f), the CD45^+^CD31^−^ control cell population decreased from 37 to 18% (Fig. 1c, e, f and Additional file 1). Furthermore, flow cytometry analysis, after 3 days of in vitro cytokine stimulation of the two distinct populations, revealed that the CD31^low^CD45^low^ cells showed an increase of CD117^+^ (18.19%), and a reduction in the classical stem/progenitor cell markers CD34^+^ (6.41%) and CD150^+^ (0.001%) (Fig. 1g); the CD31^high^CD45^+^ cells acquired these same markers after 3 days, in the following proportions: CD117^+^ (53.15%), CD34^+^ (8.32%), and CD150^+^ (1.74%) (Fig. 1g), while the majority of the control cells expressed CD117^+^ (73.27%), but there was no expression of CD34^+^ or CD150^+^ (Fig. 1g and Additional file 1). In addition, the CD31^low^CD45^low^ cell population showed an increase in the fraction of cells expressing CD34^+^CD117^+^ (from 0.22 to 23.01%) and the majority of CD31^high^CD45^+^ cell population was CD34^+^CD117^+^ (increasing from 0.11 to 61.47%). The control cell population also showed an increase in CD34^+^CD117^+^ (from 0.06 to 6.38%) (Fig. 1h and Additional file 1).

Since hematopoiesis, including erythropoiesis, is regulated by the microenvironmental niche, we further assessed the differentiation of CD31^low^CD45^low^ and CD31^high^CD45^+^ cells derived from 3-day cultures (including the CD31^−^CD45^+^ as a control cell population). To this end, we co-cultured the three-day culture cells on to bone marrow-derived, mesenchymal stromal cells (BM-MSCs) in the presence of SCF, TPO, IL-3, IL-6, Holo-Tf, and EPO for 15 days. After the co-culture, flow cytometry analysis revealed the loss of both CD31^low^CD45^low^ and CD31^high^CD45^+^ populations (Fig. 1i, left panel from the lineage-negative gate). The analysis of all live cell populations showed that most of the remaining cells were CD235b^+^ erythrocytes (81.86%), and that 1% of the total population was CD117^+^, 1.95% was CD34^+^ and 0.73% was CD34^+^CD117^+^ (Fig. 1i). In addition, there were no CD41^+^ megakaryocytic cells (not shown). The total number of cells increased from 2 × 10^5^ to 3.6 × 10^6^ after 3 days, and then 1.3 × 10^7^ by day 18 (3-day culture at + 15 days of co-culture) (Fig. 1j, histogram). The image at day 0 confirms that the majority of the lineage-negative initial cells in culture were CD31^+^CD45^+^ (Fig. 1k, left panel, at day 0). Bright-field analysis of the cells after the 3-day culture revealed that the majority of the cells were round with some heterogeneity. Occasional single cells appeared as adherent, spindle-shaped, endothelial-like cells (Fig. 1k, middle panel, day 3). The cell populations from the 18-day culture were stained with May–Grünwald Giemsa reagent (Fig. 1k, right panel day 18). Microscopic evaluation demonstrated a predominance of erythroid precursors and immature erythrocytes, i.e., pro- and basophilic erythroblasts as well as polychromatic and orthochromatic erythroblasts (arrows in Fig. 1k, right panel day 18). After differentiation, the CD31^low^CD45^low^ and CD31^high^CD45^+^ cells gave rise to cell populations that recapitulated the previously described stages of erythropoiesis starting from CD34^+^ HSCs [11]. Consistent with this, cell pellets were red, which also suggested hemoglobin synthesis during differentiation (Fig. 1h). However, in our system, none of the CD31^low^CD45^low^ cells, or any of the CD31^high^CD45^+^ cells generated the terminally-differentiated, non-nucleated erythrocytes (Fig. 1k, day18, last two panels) and neither did the control cells.

In summary, we identified a new population of circulating blood cells that express CD31^low^CD45^low^ and CD31^high^CD45^+^ that are capable of differentiation into erythroid progenitors. Our results are concordant with the data from Plein et al. [5], showing a similar population of CD31^+^CD45^+^ cells that colonize the embryo, persist into adulthood, and maintain the potential for differentiation into endothelial cells. SCF, FLT3, IL-3, IL-6, IL-16, and EPO promoted the preferential differentiation of these cells into CD31^high^CD45^+^ cells accompanied by a decrease in the CD31^low^CD45^low^ cell population. Further maturation of these CD31^low^CD45^low^ and CD31^high^CD45^+^ circulating cells generated CD235b^+^ primed erythroblasts. Finally, we identified CD31^low^CD45^low^ and CD31^high^CD45^+^ populations in peripheral blood which are capable of differentiation in vitro into erythroid progenitor cells. As mentioned previously, Bauman et al. have demonstrated that a sub-population of CD31^+^ lineage-negative, c-kit_, and Sca-1_ cells were still present in adult mouse bone marrow, exhibiting short-term erythroid cell potential and radioprotection [6]. This suggests that it may be possible that our identified cellular sub-populations, CD31^low^CD45^low^ and CD31^high^CD45^+^ are also present in bone marrow. Therefore, it would be of interest to know whether or not they are indeed present in the bone marrow, and at what frequency, and, how this might differ from the frequency found in peripheral blood. It would also be of interest to understand whether or not they are able to differentiate into erythroid progenitors under normal conditions in peripheral blood, or only in certain specific conditions such as when under stress. To answer these questions, our findings strongly support the need for further investigation by future functional studies with sorted CD31^low/high^ populations, to definitively demonstrate how these cells relate to the other stem and progenitor cells within the hematopoietic hierarchy and how they relate to previously-described EMPs.

## Supplementary Information

**Additional file 1: Figure 1.** Flow cytometric characterization of circulating CD31-CD45+ Control cells in human peripheral blood. (A) Flow cytometric analysis of fresh adult human Lin- CD31-CD45+ cell population, used as a control, from enriched hematopoietic Lin- cells peripheral blood. Representative flow cytometric analysis. (B) Representative plots showing the distribution of CD31-CD45+ CTRL cell population and immunophenotypic after three days of cytokine stimulation. (F) Cumulative data are shown as means ± SD of 3 independent experiments in the bar graph.

**Additional file 2.**

## Data Availability

All data and material generated or analyzed during this study are included in this published article [and its additional file].

## Abbreviations

HSPCs: Hematopoietic stem and progenitor cells
AGM: Aorta-gonad-mesonephros
EHT: Endothelial-to-hematopoietic transition
EMPs: Erythro-myeloid progenitors
SLAM: Signaling lymphocyte activation molecule
PBMC: Peripheral blood mononuclear cells
SDF-1: Stromal cell-derived factor 1
BM-MSCs: Bone marrow-derived mesenchymal stromal cells
