# Supplementary material for: Identification of circulating CD31+CD45+ cell populations with the potential to differentiate into erythroid cells
Source: Stem Cell Res Ther. 2021 Apr 13;12:236. doi: 10.1186/s13287-021-02311-y (PMC8042691; doi:10.1186/s13287-021-02311-y)
Supplement: Supplementary file 1 — Additional file 1: Figure 1. Flow cytometric characterization of circulating CD31-CD45+ Control cells in human peripheral blood. (A) Flow cytometric analysis of fresh adult human Lin- CD31-CD45+ cell population, used as a control, from enriched hematopoietic Lin- cells peripheral blood. Representative flow cytometric analysis. (B) Representative plots showing the distribution of CD31-CD45+ CTRL cell population and immunophenotypic after three days of cytokine stimulation. (F) Cumulative data are shown as means ± SD of 3 independent experiments in the bar graph. [file 13287_2021_2311_MOESM1_ESM.pdf]

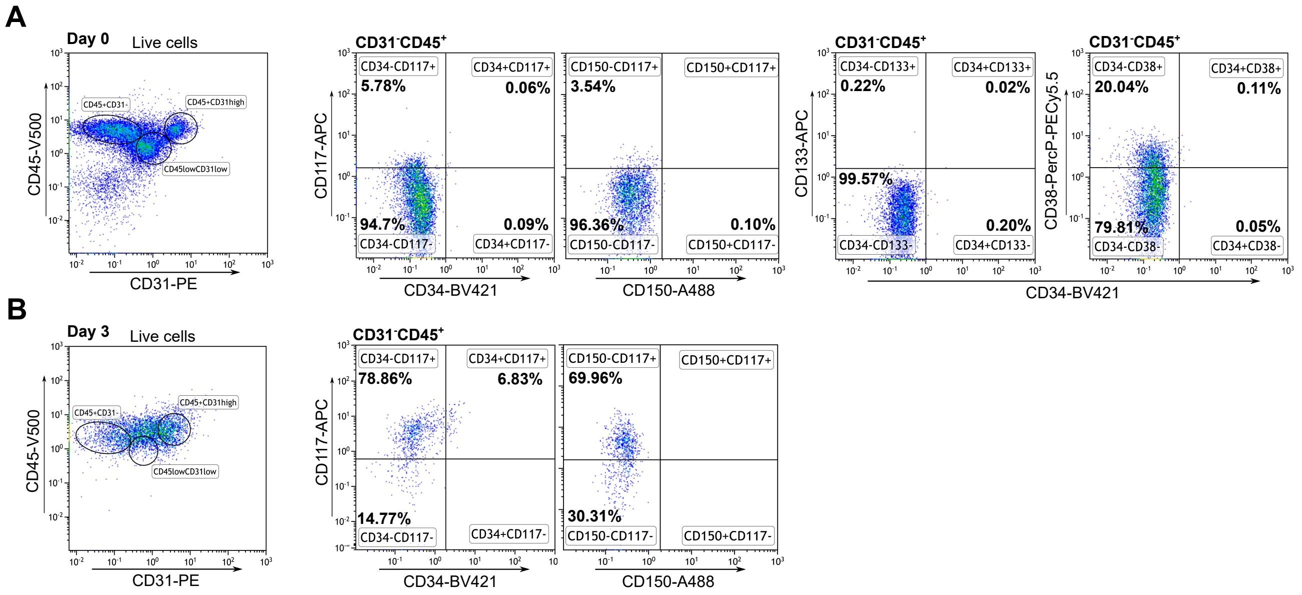

Figure 1. Flow cytometric characterization of circulating CD31$^-$CD45$^+$ Control cells in human peripheral blood. (A) Flow cytometric analysis of fresh adult human Lin$^-$ CD31$^-$CD45$^+$ cell population, used as a control, from enriched hematopoietic Lin$^-$ cells peripheral blood. Representative flow cytometric analysis. (B) Representative plots showing the distribution of CD31$^-$CD45$^+$ CTRL cell population and immunophenotypic after three days of cytokine stimulation. (F) Cumulative data are shown as means ± SD of 3 independent experiments in the bar graph.
