# Supplementary material for: Identification of circulating CD31+CD45+ cell populations with the potential to differentiate into erythroid cells
Source: Stem Cell Res Ther. 2021 Apr 13;12:236. doi: 10.1186/s13287-021-02311-y (PMC8042691; doi:10.1186/s13287-021-02311-y)
Supplement: Supplementary file 2 — Additional file 2. [file 13287_2021_2311_MOESM2_ESM.docx]

**Supplemental Materials and Methods**

**Lin^-^ Cell Isolation**

Whole blood was collected from adult healthy donors at the Transfusion Medicine Blood Bank of the National Institutes of Health. Signed informed consent was obtained in accordance with the NIH Institutional Review Board (IRB number 99-CC-1068). Human Lin^-^ mononuclear cells were isolated by negative selection using the RosetteSep™ Human Hematopoietic Progenitor Cell Enrichment Cocktail Kit in combination with RosetteSep™ Human Monocyte Depletion Cocktail Kit (Stemcell Technologies Inc., [Vancouver, Canada](https://www.google.it/search?biw=1366&bih=643&q=Vancouver&stick=H4sIAAAAAAAAAOPgE-LSz9U3MMmurCw2UOIAsS0MMoy0tLKTrfTzi9IT8zKrEksy8_NQOFYZqYkphaWJRSWpRcUAtJVm70QAAAA&sa=X&sqi=2&ved=0ahUKEwj_z9OsrdTMAhViCsAKHUXACl8QmxMImQEoATAQ)), from healthy donors standard whole blood preparation, following the manufacturer’s instructions. Specifically, lineage-depleted whole blood samples were obtained using a cocktail of monoclonal antibodies directed against CD2, CD3, CD14, CD16, CD19, CD24, CD36, CD56, CD66b, and glycophorin A, on red blood cells (RBCs) in combination with CD36 to deplete monocytes. Lysis of residual RBCs with Ammonium Chloride Solution (Stemcell Technologies Inc.) was performed prior to flow cytometry analysis and/or to subsequent assays.

**Three-day in vitro cytokine stimulation of CD31^+^CD45^+^ cells**

CD31^+^CD45^+^ cells were stimulated with SCF, FLT3, TPO, (50 ng/ml), CXCL12, IL-3, IL-6, (each at 10 ng/mL), and EPO (2 mU/ml) (4ng/ml) (PeproTech, London, UK) (based on our previous studies^1-4^ and preliminary experiments) for three days in complete culture medium (alpha MEM containing 10% FBS) in six well plates (Greiner CELLSTAR dish, Sigma-Aldrich, [Saint Louis, MO,](https://www.google.it/search?biw=1517&bih=714&q=St.+Louis&stick=H4sIAAAAAAAAAOPgE-LUz9U3sLC0SK5U4gAxzcoryrW0spOt9POL0hPzMqsSSzLz81A4VhmpiSmFpYlFJalFxQDMHhGVQwAAAA&sa=X&ved=0ahUKEwjGq8-BstTMAhVIP5oKHWu_BWUQmxMIhgEoATAQ) USA) (2x10^5^ cells/well). After three days, CD31^+^CD45^+^ cells were washed two times with PBS (Life Technologies, Carlsbad, CA, USA) then counted and analyzed for the presence of cell surface antibodies by flow cytometry analysis and/or subsequent assays, as described below.

**CD31^+^CD45^+^ three-day stimulated cells co-cultured on BM-MSC**

 Human bone marrow–derived MSC (BM-MSC; Cambrex BioScience) were propagated in Dulbecco modified Eagle medium, low glucose (Invitrogen Corporation, Carlsbad, CA, USA) containing 10% fetal bovine serum (FBS) and 1% penicillin-streptomycin. MSCs were tested positive for CD90, CD44, CD29, CD105, and CD166; negative for CD45, CD31, CD117, CD34, Flk1, Flt1, CD13, and CXC chemokine receptor (CXCR4) (data not shown).^4^ For co-culture with CD31^+^CD45^+^ three-day stimulated cells, hBMSCs were irradiated (1500 cGy) before being transferred to plates. CD31^+^CD45^+^ three-day stimulated cells were transferred onto irradiated hBMSCs layers and cultured with differentiation media composed of IMDM (Invitrogen) supplemented with 10% FBS. To support the development of erythroid lineages, we added the following cytokines: SCF, TPO, (5 ng/ml), IL-3, IL-6, (each at 3 ng/mL), EPO (2 mU/ml) (4 ng/ml) (PeproTech, London, UK), Hydrocortisone (10e-6 M) and Holotrasferrin (200 µg/ml) (Sigma, St Louis, MO), based on our previous studies^1-4^ and preliminary experiments. The medium was changed every two to three days and cells were cultured for a total of 15 days, after which the CD31^+^CD45^+^ differentiated cells were harvested, then analyzed by flow cytometry, immunofluorescence, and histochemistry

**Flow Cytometric Analysis**

Flow cytometric analysis was performed immediately after Lin^-^ cell isolation. Then three days after cytokine cocktail stimulation in culture and 15 days after co-culture with MSCs, in the presence of cytokines, the cells were harvested and analyzed for the presence of cell surface antibodies, according to standard protocols. Cells were stained with various conjugated anti-human antibodies and in different combinations, including: CD45-V500, CD31-PE, CD117-APC, CD34-BV421, CD150-A488, CD133-APC, CD38-PeCP/PECy55, CD14-FITC, CD41-FITC, CD3-FITC, CD19- BV421, and Glycophorin B-PE (all from BD Bioscience), for 30 minutes in the dark in a cell staining buffer (0.5 % BSA/PBS). After the addition of DAPI (1 μg ml^−1^ Thermo Fisher Scientific), to exclude dead cells, the different cell preparations were acquired using MoFlo Astrios EQ high speed cell sorter (Beckman Coulter). Flow data analysis was performed using Summit software V6.3.016900 (Beckman Coulter).

**Immunofluorescence and Histochemistry**

Cells from three and 15 days post co-culture, were stained with May Grumwald Giemsa (Carlo Erba, Milano, Italy) or with anti–human CD31/PECAM-1 mAb (R&D Systems), and anti–human CD45 (MyBioSource), and incubated in the dark with secondary fluorochrome conjugated antibodies goat anti–mouse Alexa Fluor 488 or goat anti–rabbit Alexa Fluor 555 (both from Invitrogen) and then analyzed by fluorescent microscope (CFC425 C, Leica, Milton Keynes, UK).

**Statistical analyses**

Statistical analyses were performed using the GraphPad Prism Software 8.0 and data are expressed as mean values ± standard deviation (SD).

References

1. [Ciraci E, Della Bella S, Salvucci O, et al. Adult human circulating CD34⁻Lin⁻CD45⁻CD133⁻ cells can differentiate into hematopoietic and endothelial cells.](https://pubmed.ncbi.nlm.nih.gov/21715308/) Blood. 2011 Aug 25;118(8):2105-15.
2. Naoya Uchida, Juan J. Haro-Mora, et al. [Efficient generation of β-globin-expressing erythroid cells using stromal cell-derived induced pluripotent stem cells from patients with sickle cell disease](https://www.ncbi.nlm.nih.gov/pmc/articles/PMC5330841/) Stem Cells. 2017 Mar; 35(3): 586–596.
3. Shen J, Zhu Y, Lyu C, et al. [Sequential cellular niches control the generation of enucleated erythrocytes from human pluripotent stem cells.](https://pubmed.ncbi.nlm.nih.gov/31197070/) Haematologica. 2020 Jan 31;105(2):e48-e51.
4. Salvucci O, Maric D, Economopoulou M, rt al. [EphrinB reverse signaling contributes to endothelial and mural cell assembly into vascular structures.](https://pubmed.ncbi.nlm.nih.gov/19411631/).Blood. 2009 Aug 20;114(8):1707-16.
